# The mysterious case of the *C. elegans* gut granule: death fluorescence, anthranilic acid and the kynurenine pathway

**DOI:** 10.3389/fgene.2013.00151

**Published:** 2013-08-07

**Authors:** Cassandra Coburn, David Gems

**Affiliations:** Institute of Healthy Ageing, and Department of Genetics, Evolution and Environment, University College LondonLondon, UK

**Keywords:** aging, *C. elegans*, death fluorescence, gut granule, kynurenine, lipofuscin, organismal death, tryptophan

## Abstract

Gut granules are lysosome-like organelles with acidic interiors that are found in large numbers within the intestine of the nematode *Caenorhabditis elegans*. They are particularly prominent when viewed under ultraviolet light, which causes them to emit intense blue fluorescence. Yet the function of these large and abundant organelles in this heavily-studied model organism remains unclear. One possibility is that they serve as storage organelles, for example of zinc. A new clue to gut granule function is the identification of the blue fluorescent material that they contain as a glycosylated form of anthranilic acid, which is derived from tryptophan by action of the kynurenine pathway. This compound can also serve a surprising role as a natural, endogenous marker of organismal death.

## THE GUT GRANULE: AN ENIGMATIC NEMATODE ORGANELLE

Despite decades of research on the nematode *Caenorhabditis elegans*, it still contains many hidden secrets. One such is the function of the prominent organelles known as gut granules, which are numerous in the intestinal cells of nematodes throughout the suborder Rhabditina (Chitwood and Chitwood, 1950). A striking feature of gut granules is the blue fluorescence that they emit under ultraviolet light (Klass, 1977; Gerstbrein et al., 2005). Clues to gut granule function include their acidic interior and capacity for endocytosis (Clokey and Jacobson, 1986; Hermann et al., 2005), both lysosome-like features (though gut granules are much bigger than normal lysosomes). This and the fluorescent material within identify gut granules as lysosome-like organelles (LROs; Hermann et al., 2005; Bernabucci et al., 2012), akin to pigment-containing melanosomes in mammals and eye pigment granules in *Drosophila* (Raposo and Marks, 2007). Thus, the identity of the blue fluorescent substance could provide a key to understanding gut granule function.

One suggestion is that the source of gut granule fluorescence is lipofuscin, a complex molecular waste production that accumulates within lysosomes in aging mammalian cells (Jung et al., 2007). Lipofuscin can contain Schiff bases, which have similar spectral similarities to the worm blue fluorescence (Fletcher et al., 1973; Klass, 1977). Consistent with this, blue fluorescence levels increase in aging worm populations (Klass, 1977; Davis et al., 1982; Gerstbrein et al., 2005). Another idea, derived from studies of *C. elegans* Flu mutants with altered fluorescence color and intensity, is that the blue fluorescence emanates from L-tryptophan-derived metabolites called kynurenines (Babu, 1974).

Over the years the lipofuscin interpretation has been favored (see e.g., Gill, 2006; Masse et al., 2008; Fujii et al., 2009; Jain et al., 2009; Minniti et al., 2009), perhaps because of the good fit with the theory that aging is caused by accumulation of molecular damage. Unfortunately, this interpretation (i.e., that the blue fluorescent substance is lipofuscin) is not the correct one. According to recent chemical analysis, the fluorescent substance within gut granules is a kynurenine pathway product, anthranilic acid (AA) glucosyl ester (Coburn et al., 2013), consistent with the proposal of P. Babu and S. S. Siddiqui so many years ago (Babu, 1974; Bhat and Babu, 1980; Siddiqui and Babu, 1980). This chemical identification was effected by comparing wild-type worms with *glo-1* mutants, which lack gut granules (Hermann et al., 2005). Whether or not lipofuscin exists in *C. elegans* remains an open question. Thus, *C. elegans* gut granules contain large quantities of AA. But what it is there for? Here, one may seek clues from kynurenine pathway action in mammals.

## THE KYNURENINE PATHWAY AND NEURODEGENERATION

In mammals, the kynurenine pathway generates a variety of important molecules, including the co-factor nicotine adenine dinucleotide (NAD) and the neurotransmitter serotonin. Around 95% of tryptophan (the rarest essential amino acid) is consumed by this pathway (Vecsei et al., 2013). Although discovered over 150 years ago, the action of the kynurenine pathway’s intermediate metabolites, known as kynurenines, has until recently been relatively little studied (Schwarcz et al., 2012). One role of kynurenines is in modulating CNS excitability (Perkins and Stone, 1982; Hilmas et al., 2001; Vecsei et al., 2013). For example, the kynurenine quinolinic acid stimulates *N*-methyl-D-aspartate (NMDA) receptors (Stone and Perkins, 1981; Schwarcz et al., 2012), while kynurenic acid antagonizes all excitatory amino acid receptors.

Kynurenine pathway dysregulation has been implicated in neurological disorders, including Huntington’s, Alzheimer’s, and Parkinson’s disease, multiple sclerosis, and epilepsy (Vecsei et al., 2013) as well as in neurodegeneration caused by acute insults, such as ischemia and excitotoxicity (Stone et al., 2012). Excitotoxic neurodegeneration is caused by release of high levels of excitatory neurotransmitters, which trigger an influx of calcium ions after depolarization (Rothman and Olney, 1987). Thus, calcium can act as a second messenger, triggering the initiation of necrotic cell death (Rothman and Olney, 1995). The kynurenine quinolinic acid can act as an excitotoxin: levels increase following ischemia, and correlate with increased neurodegeneration (Saito et al., 1993). Thus, one of the ways in which kynurenines may contribute to neurodegenerative disease is by inducing excitotoxic neurodegeneration.

## THE KYNURENINE PATHWAY IN *C. elegans*

**FIGURE 1:**
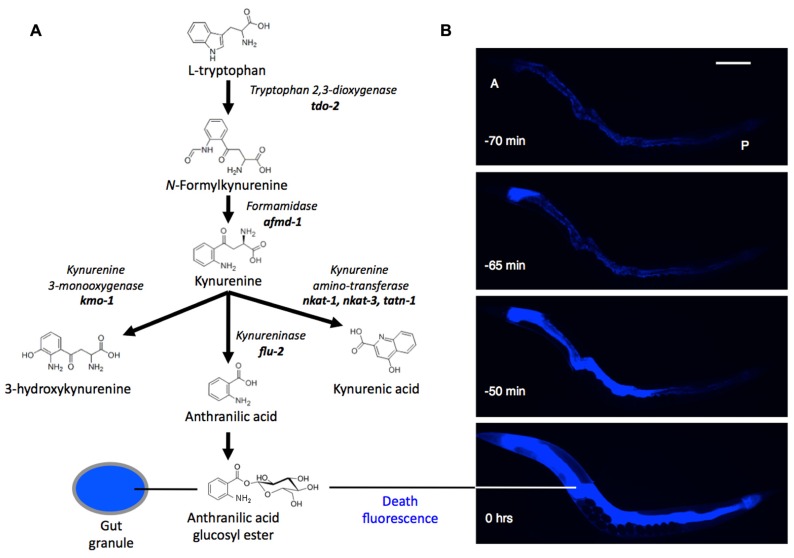
**(A)** Synthesis of anthranilic acid by the kynurenine pathway. **(B)** Death fluorescence in young adult *C. elegans* killed with a heated wire (DAPI filter). During death fluorescence the pattern of fluorescence changes from punctate (issuing from gut granules) to diffuse, and much brighter. A, P, anterior and posterior ends of intestine. Time is relative to peak fluorescence. Scale bar, 200 μm.

Is there a link between kynurenines and aging, particularly neurodegeneration, in *C. elegans*? Very little is known about the biology of kynurenines in nematodes. One exception relates back to gut granules: among the Flu mutants alluded to previously, altered intestinal fluorescence (Flu) phenotypes can arise from mutations affecting kynurenine pathway enzymes. For example, *flu-1* mutants, which show an altered, bluish-purple gut granule fluorescence, have reduced kynurenine-3-hydroxylase activity (Siddiqui and Babu, 1980), and *flu-2* mutants, which show a dull green fluorescence, have reduced kynureninase (Bhat and Babu, 1980; **Figure 1A**). The *C. elegans* genome contains homologs of genes encoding these two enzymes in the vicinity of the *flu-1* and *flu-2* loci: a kynurenine hydroxylase, R07B7.5, and a kynureninase C15H9.7, respectively (Altschul et al., 1990; Kanehisa, 2012). Other predicted kynurenine pathway genes are also present in *C. elegans* (van der Goot and Nollen, 2013).

In *Drosophila* genetic and pharmacological inhibition of the kynurenine pathway enzyme tryptophan 2,3-dioxygenase (TDO) extends longevity (Oxenkrug, 2010; Oxenkrug et al., 2011). This suggests that kynurenines may contribute to pathologies of aging; however, whether this is true in *C. elegans* remains uncertain. Here RNAi knock-down of *tdo-2* reduced the toxicity of α-synuclein aggregation in a Parkinson’s disease model, and increased lifespan (van der Goot et al., 2012). However, these effects proved to be caused by increased levels of tryptophan rather than altered levels of kynurenines (van der Goot et al., 2012; for a detailed review of the kynurenine pathway and aging see van der Goot and Nollen, 2013). *tdo-2* RNAi also abrogates gut granule fluorescence in the worm (Coburn et al., 2013).

Kynurenines also play a startling role in the biology of death in *C. elegans*. As they die, worms emit a dramatic burst of blue AA fluorescence (Coburn et al., 2013; **Figure 1B**). This *death fluorescence* typically occurs in an anterior to posterior wave that courses along the intestine, and is seen in both young worms subjected to lethal injury, and worms dying peacefully of old age. Death fluorescence is a somewhat eerie phenomenon in that it renders visible the passage of death through the semi-transparent body of the worm as a spectral blue glow.

Death fluorescence is promoted by the calpain–cathepsin necrotic cell death cascade. In this cascade, intracellular Ca^2^^+^ levels rise, activating Ca^2^^+^-dependent calpains (cysteine proteases; Yamashima et al., 1996). These cause lysosomal lysis, leading to cytosolic acidosis and the destructive release of lysosomal cathepsin proteases (Yamashima and Oikawa, 2009). Mutational attenuation of this cascade often reduces death fluorescence (Coburn et al., 2013). Moreover, the intercellular propagation of death fluorescence (and, probably, necrosis) is dependent upon the innexin gap junction INX-16, reminiscent of the spread of excitotoxic neuronal death from one cell to another in mammals. How exactly the necrotic cascade leads to increased AA fluorescence remains unclear, but one possibility is that it reflects AA fluorescence dequenching as it is released from the gut granules upon organellar lysis.

## POSSIBLE FUNCTIONS OF ANTHRANILATES AND GUT GRANULES IN *C. elegans*

The significance of AA concentrated within gut granules remains unclear. One possibility is that glycosylation of AA contributes to its accumulation; in *Arabidopsis*, glycosylation by UDP-glucosyltransferases promotes AA accumulation by increasing compound stability (Quiel and Bender, 2003). Regarding function, one possibility is that AA serves a protective role. In mammals kynurenines can contribute to immune function (Munn et al., 1998; Fallarino et al., 2002; Piscianz et al., 2011). Moreover, AA can inhibit growth of bacterial pathogens, e.g., *Legionella pneumophila* (Sasaki et al., 2012). Thus, AA might have antibiotic properties in *C. elegans*, in which case gut granules could serve as a store of anti-bacterial agents in the event of pathogen attack. This suggests a broader role for gut granules: that of chemical weapons depots for *C. elegans *in their war against the diverse pathogens that beset them in their natural environment (Felix and Braendle, 2010). This could also explain the presence of gut granules in the intestine, the site most likely to experience pathogenic invasion in *C. elegans* (Hodgkin and Partridge, 2008). Another possibility, suggested by similarities between gut granules and melanosomes, is that they are photoprotective. AA fluorescence (peak λ_ex_/λ_em_ 340 nm/430 nm) entails the conversion of damaging UV light to relatively harmless visible light, and so may protect against UV damage.

The large size of gut granules relative to ordinary lysosomes is consistent with function as a storage organelle. Moreover, gut granules are the major site of storage of zinc in the worm (Roh et al., 2012). Interestingly, when zinc levels are high, gut granule morphology changes, becoming bilobed, including an apparently non-acidic compartment in which zinc is concentrated. How distribution of zinc and AA compares in such bilobed gut granules remains to be established. It is also notable that both metal toxicity and kynurenines are determinants of neurodegenerative disease. Gut granules also stain with the lipid staining vital dye Nile red; however, results of careful analysis imply that this does not reflect the presence of lipid within gut granules (O’Rourke et al., 2009).

Ultimately, the role in *C. elegans* biology of gut granules and the AAs they contain remains obscure and a topic for future investigation. But we now know at least that the fluorescence of these prominent organelles issues from AA glucosyl esters, rather than lipofuscin – removing one reason for believing that worm aging is caused by accumulation of molecular damage, and opening the way for alternatively theories (Gems and de la Guardia, 2012). And we know that gut granule decay contributes to a wave of intestinal necrosis accompanied by a burst of blue anthranilate fluorescence, which serves as a useful marker for organismal death in *C. elegans*.

## Conflict of Interest Statement

The authors declare that the research was conducted in the absence of any commercial or financial relationships that could be construed as a potential conflict of interest.
